# Associations Between Lifestyle Factors and Constipation Among Survivors After the Great East Japan Earthquake: A 9-year Follow-up Study

**DOI:** 10.2188/jea.JE20220284

**Published:** 2024-04-05

**Authors:** Moeka Harada, Nobuyo Tsuboyama-Kasaoka, Yuki Yonekura, Haruki Shimoda, Akira Ogawa, Seiichiro Kobayashi, Kiyomi Sakata, Nobuo Nishi

**Affiliations:** 1 National Institutes of Biomedical Innovation, Health and Nutrition, Osaka, Japan; 2 St. Luke’s International University, Tokyo, Japan; 3 Iwate Medical University, Iwate, Japan

**Keywords:** Great East Japan Earthquake, constipation, lifestyle factors, dietary intake

## Abstract

**Background:**

Disaster survivors experience deterioration in lifestyles and an increase in constipation. After the Great East Japan Earthquake in 2011, some survivors were evacuated for a long term, even after moving to temporary housing and public reconstruction housing. However, annual changes in constipation and the association between lifestyles and constipation among the survivors are still unknown.

**Methods:**

Overall, 9,234 survivors aged 18 years or older participated in this 9-year follow-up survey after the disaster. Information about the prevalence of constipation and lifestyle factors (diet, physical activity, and mental health) was collected using a self-reported questionnaire. Their dietary intake was categorized into the following two dietary patterns: prudent (fish and shellfish, soybean products, vegetables, fruits, and dairy products) and meat (meat and eggs). Odds ratios for constipation according to lifestyle factors were calculated using a generalized linear mixed model.

**Results:**

In women, the prevalence of constipation was the highest at baseline (8.7%) and remained around 5% afterward. In both men and women, older age, poor mental health, and poor physical activity were significantly associated with higher odds ratios of constipation. Moreover, a lower frequency of meals and a lower prudent dietary score were significantly associated with women’s constipation.

**Conclusion:**

The prevalence of constipation was the highest at baseline and remained around 5% in women. Lifestyle factors, such as poor mental health, physical inactivity, and low frequency of meals were associated with constipation. Our findings suggest continuous support for the survivors with constipation for medium- to long-term after disasters.

## INTRODUCTION

Constipation has been reported to be associated with increased mortality and cardiovascular events. Individuals with constipation have 12% higher all-cause mortality (hazard ratio [HR] 1.12; 95% confidence interval [CI], 1.11–1.13), 11% higher incidence of coronary heart disease (HR 1.11; 95% CI, 1.08–1.14), and 19% higher incidence of ischemic stroke (HR 1.19; 95% CI, 1.15–1.22) compared to those without constipation.^1^ In addition, constipation has been identified as one of the factors for worsening health-related quality of life. The impact of constipation on health-related quality of life is comparable with that of other health conditions, including chronic obstructive pulmonary disease, diabetes, and depression.^2^ As the health-related quality of life decreases after disasters,^3^ constipation in disaster survivors should be addressed.

In March 2011, the Great East Japan Earthquake (GEJE) caused severe damage, and up to 470,000 people were forced to evacuate.^4^ One month after the GEJE, 10% of evacuees complained of constipation as the most common gastrointestinal symptom in an emergency shelter.^5^ Furthermore, even 9 years after the earthquake, more than 48,000 people remained evacuated (eg, living in temporary housing and public reconstruction housing).^6^ The constipation was expected to be prolonged due to the extended evacuation. However, the persistence of constipation among survivors of GEJE is still unknown.

Risk factors for health problems that are reported to be associated with evacuation include disruption of lifelines, deterioration in the quantity and quality of food, lack of exercise, increased psychological stress, sleep disorders, lack of social networking, and increased rates of smoking.^7^^–^^13^ Among these, risk factors for constipation in normal periods include deteriorating lifestyles, such as diet, physical activity, and mental health.^14^^–^^17^ Moreover, these lifestyle factors deteriorate among survivors after disasters.^7^^–^^10^ For example, survivors living in temporary housing had low dietary intakes of fruits and vegetables and soybean products.^10^ Additionally, evacuated survivors at accommodations other than their own homes had low physical activity^8^ and poor mental health.^9^ Therefore, constipation among the survivors is expected to be associated with a worsening lifestyle. Thus, improving lifestyle factors in evacuees may control constipation during evacuation and improve survival rates among the survivors. However, to the best of our knowledge, no studies have clarified this result.

Therefore, this study investigated the annual changes in constipation after GEJE, and to clarify the association between lifestyle factors and constipation among survivors in a 9-year follow-up.

## METHODS

### Study participants

**Figure 1.**
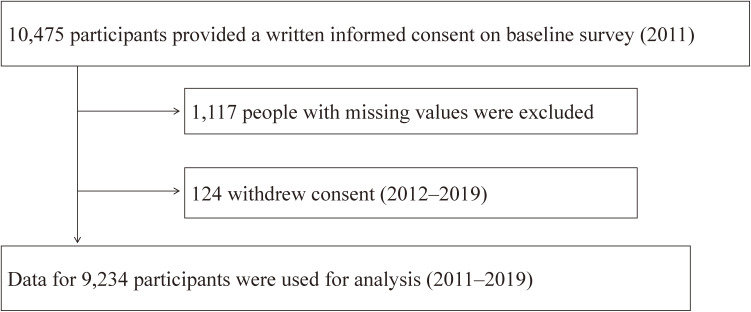
Flow diagram of study participants in the present analysis

This study is based on the Research project for prospective Investigation of health problems Among Survivors of the 2011 GEJE and Tsunami (RIAS) study.^9^^,^^18^ This population-based longitudinal cohort study started in 2011 and repeated health checkups with assessment using a questionnaire every year. The participants were residents aged 18 years or older in Rikuzentakata city, Kamaishi city, Yamada town, and Otsuchi town. The flow diagram of study participants in the present analysis is shown in Figure 1. The analysis included 9,234 individuals who participated in 2011 (baseline) survey, and their data obtained from 2011–2019 surveys were used for the analysis. Thus, the participants’ data with at least baseline data, not limited to those with complete data for 9 years.

### Variables

Constipation was one of 25 subjective symptoms in the self-reported questionnaire. We also recorded their age, dietary intake, mental health, physical activity, and housing situation. Regarding dietary intake, we asked about the frequency of meals per day and the frequency of food intake during the previous several days. The food groups that we surveyed were: staple food (rice, bread, and noodles), meat, fish and shellfish, eggs, soybean products (tofu and *natto* [fermented soybeans]), vegetables, fruit, and dairy products (milk, yogurt, and cheese).^19^ The mental health was assessed using the Kessler-6-item Psychological Distress (K6) Scale.^20^^,^^21^ The validated physical activity questionnaire comprised three questions: the frequency of performing housework and occupation, all physical activities, the frequency of leaving their house, and walking duration per day.^22^

Food groups were categorized based on the participants’ dietary patterns, as described by Nishi et al (2013).^23^ The prudent dietary pattern comprised fish and shellfish, soybean products, vegetables, fruits, and dairy products. The meat dietary pattern included meat and eggs. A score for each dietary pattern was calculated according to the frequency of intake of each food group for all participants. The response categories were the following: none (<once a day) = 0 point, once a day = 1 point, twice a day = 2 points, three times a day = 3 points, and four times or more a day = 4 points. Thus, the range of prudent dietary score was calculated as 0–20 and the meat dietary score as 0–8. Total points of mental health ranged from 0 to 24. In this study, good and poor mental health were categorized as scores from 0 to 4 and >5, respectively. Total scores for the physical activity ranged from 1 to 15. A score of ≥13.5, equivalent to 23 METs hour per week, was categorized as good physical activity, and a score of <13.5 was classified as poor physical activity.^22^ The current housing situation was categorized as temporary housing (prefabricated temporary housing and emergency shelter) and non-temporary housing.

### Statistical analysis

Multivariate logistic regression analyses were performed by sex. The dichotomous dependent variable was constipation (presence or absence [reference]). The independent variables were age, frequency of meals, prudent dietary score, meat dietary score, mental health (good [reference] or poor), physical activity (good [reference] or poor), housing situation (temporary housing or non-temporary housing [reference]), residential area (Rikuzentakata city [reference], Yamada town, Otsuchi town, and Kamaishi city), and year of survey (the first year [reference] or the second year through the ninth year). Because repeatedly measured data were obtained from the participants, we used a generalized linear mixed model (GLMM) with a logit link. GLMM allows for longitudinal data analysis with missing data. Results were presented as odds ratios (ORs) with 95% CIs. All analyses were conducted using IBM SPSS Statistics version 28.0 (IBM Corp., Armonk, NY, USA). *P*-values less than 0.05 were considered statistically significant.

### Ethics approval

This study was approved by the ethics committees of the National Institutes of Biomedical Innovation, Health and Nutrition (approval number: KENEI99) and Iwate Medical University (approval number: H23-69).

## RESULTS

The characteristics of participants by sex are shown in Table 1. Among 9,234 participants, 38.9% were men, and 61.1% were women. The mean of age was 61.9 (standard deviation [SD], 14.5) years for men and 59.8 (SD, 14.7) years for women.

**Table 1.** Baseline characteristics of participants

	Men			Women		
	
	Total (*n* = 3,592)	Constipation		Total (*n* = 5,642)	Constipation	
	
		Present (*n* = 175)	Absent (*n* = 3,417)		Present (*n* = 491)	Absent (*n* = 5,151)
Age, years, mean (SD)	61.9 (14.5)	68.5 (13.8)	61.6 (14.5)	59.8 (14.7)	60.2 (16.6)	59.8 (14.5)
Frequency of meals, mean (SD)	2.9 (0.3)	2.9 (0.3)	2.9 (0.3)	3.0 (0.3)	2.9 (0.3)	3.0 (0.3)
Prudent dietary score, mean (SD)	7.3 (2.8)	7.4 (2.6)	7.3 (2.8)	7.9 (2.7)	7.8 (2.9)	7.9 (2.7)
Meat dietary score, mean (SD)	2.0 (1.1)	1.8 (1.1)	2.0 (1.1)	1.9 (1.0)	1.9 (1.1)	1.9 (1.0)
Mental health, *n* (%)						
Good [K6 0–4]	2,274 (100.0)	81 (3.6)	2,193 (96.4)	2,975 (100.0)	155 (5.2)	2,820 (94.8)
Poor [K6 ≥5]	1,318 (100.0)	94 (7.1)	1,224 (92.9)	2,667 (100.0)	336 (12.6)	2,331 (87.4)
Physical activity, *n* (%)						
Good [≥23 METs h/w]	1,404 (100.0)	49 (3.5)	1,355 (96.5)	1,826 (100.0)	113 (6.2)	1,713 (93.8)
Poor [<23 METs h/w]	2,188 (100.0)	126 (5.8)	2,062 (94.2)	3,816 (100.0)	378 (9.9)	3,438 (90.1)
Housing situation, *n* (%)						
Non-temporary housing	2,041 (100.0)	85 (4.2)	1,956 (95.8)	3,125 (100.0)	249 (8.0)	2,876 (92.0)
Temporary housing	1,551 (100.0)	90 (5.8)	1,461 (94.2)	2,517 (100.0)	242 (9.6)	2,275 (90.4)
Residential area, *n* (%)						
Rikuzentakata city	1,680 (100.0)	84 (5.0)	1,596 (95.0)	2,675 (100.0)	254 (9.5)	2,421 (90.5)
Yamada town	1,154 (100.0)	48 (4.2)	1,106 (95.8)	1,671 (100.0)	114 (6.8)	1,557 (93.2)
Otsuchi town	676 (100.0)	36 (5.3)	640 (94.7)	1,144 (100.0)	102 (8.9)	1,042 (91.1)
Kamaishi city	82 (100.0)	7 (8.5)	75 (91.5)	152 (100.0)	21 (13.8)	131 (86.2)

K6, Kessler 6-item Psychological Distress Scale; SD, standard deviation.

Table 2 shows the annual change in the prevalence of constipation. In the first year (the year of GEJE), constipation was observed in 4.9% of men and 8.7% of women. The prevalence by age group (2011) was as follows: in men: 0.9% (20s), 3.5% (30s), 3.4% (40s), 2.8% (50s), 4.0% (60s), 5.5% (70s), and 15.2% (over 80s); in women: 13.4% (20s), 10.4% (30s), 7.8% (40s), 7.9% (50s), 6.4% (60s), 10.3% (70s), and 13.8% (over 80s). The 9-year follow-up data indicated that the proportions of constipation were between 4.0% and 5.8% in men. Still, the ratio was the highest (8.7%) in the first year and remained around 5% afterward in women.

**Table 2.** Prevalence of participants with constipation

Year	Men		Women	
	
	Total, *n*	Constipation, %	Total, *n*	Constipation, %
1st year (2011)	3,592	4.9	5,642	8.7
2nd year (2012)	2,511	4.2	4,195	7.5
3rd year (2013)	2,285	4.8	3,880	7.6
4th year (2014)	2,256	5.8	3,739	7.4
5th year (2015)	2,145	5.4	3,669	7.8
6th year (2016)	2,034	4.0	3,563	4.7
7th year (2017)	1,955	4.1	3,442	5.5
8th year (2018)	1,881	4.0	3,313	4.4
9th year (2019)	1,774	4.7	3,211	5.7

Table 3 shows the ORs of constipation analyzed by GLMM with a logit link. In women, the ORs of constipation were higher than unity in the 3^rd^–5^th^ year, but they fell below unity after the 6^th^ year (2016). Significantly positive associations were observed between constipation and old age, poor mental health, and poor physical activity in both men and women. In women, the frequency of meals and the prudent dietary score were significantly negatively associated with constipation.

**Table 3.** Odds ratios and 95% CIs for constipation on GLMM with logit link

Variables^a^	Men	Women
	
	OR (95% CI)	OR (95% CI)
Age, years	1.06 (1.05–1.07)	1.01 (1.01–1.02)
Frequency of meals, per a day	0.98 (0.72–1.34)	0.71 (0.59–0.86)
Prudent dietary score, points	0.99 (0.96–1.02)	0.98 (0.96–0.998)
Meat dietary score, points	0.97 (0.90–1.05)	1.02 (0.98–1.07)
Mental health		
Good [K6 0–4]	1.00 (Ref)	1.00 (Ref)
Poor [K6 ≥5]	2.01 (1.72–2.34)	1.97 (1.80–2.15)
Physical activity		
Good [≥23 METs h/w]	1.00 (Ref)	1.00 (Ref)
Poor [<23 METs h/w]	1.46 (1.26–1.70)	1.26 (1.14–1.40)
Housing situation		
Non-temporary housing	1.00 (Ref)	1.00 (Ref)
Temporary housing	1.10 (0.90–1.34)	1.07 (0.95–1.21)
Residential area		
Rikuzentakata city	1.00 (Ref)	1.00 (Ref)
Yamada town	0.79 (0.64–0.98)	0.76 (0.66–0.87)
Otsuchi town	0.98 (0.77–1.24)	0.86 (0.74–0.996)
Kamaishi city	1.20 (0.71–2.04)	1.53 (1.14–2.05)
Year		
1st year (2011)	1.00 (Ref)	1.00 (Ref)
2nd year (2012)	0.92 (0.74–1.15)	0.97 (0.85–1.11)
3rd year (2013)	1.03 (0.79–1.32)	1.10 (0.94–1.28)
4th year (2014)	1.24 (0.96–1.60)	1.06 (0.90–1.25)
5th year (2015)	1.05 (0.80–1.37)	1.06 (0.89–1.25)
6th year (2016)	0.77 (0.57–1.02)	0.64 (0.53–0.77)
7th year (2017)	0.68 (0.50–0.92)	0.72 (0.59–0.87)
8th year (2018)	0.65 (0.47–0.89)	0.59 (0.48–0.72)
9th year (2019)	0.84 (0.62–1.14)	0.74 (0.61–0.91)

CI, confidence interval; GLMM, generalized linear mixed model; K6, Kessler 6-item Psychological Distress Scale; OR, odds ratio.

^a^All variables listed in the table were adjusted multivariately.

## DISCUSSION

The present study examined the annual changes in constipation and the association between lifestyle factors and constipation among survivors of the GEJE in a 9-year follow-up study. In men and women, constipation was significantly associated with older age, poor mental health, and poor physical activity. In women, a lower frequency of meals and lower prudent dietary scores were significantly associated with constipation.

### Constipation after disaster

The prevalence of constipation among Japanese adults in 2010—the year before the GEJE—was 2.5% in men and 5.1% in women according to Ministry of Health, Labour and Welfare.^24^ Based on the data of the present study, it is possible that the prevalence of constipation increased after the GEJE in both men and women. However, it should be noted that the age distribution and the survey area of the present study were different from those reported by the Ministry of Health, Labour and Welfare. The constipation rates in the present study in terms of age groups were higher in 2011 than in 2010^24^ for almost all age groups. Moreover, a previous study compared age-adjusted prevalence rates of constipation before and after the GEJE in a survey of affected areas in Miyagi Prefecture.^25^ It reported that the relative ratio was 2.3 times higher after the GEJE than in normal times in general population.^25^ Furthermore, it is necessary to consider whether the area of this study was an area with a particularly high prevalence of constipation before the GEJE; however, to the best of our knowledge, there were no reports on the prevalence of constipation in the area of this study. Therefore, we discussed the area-related characteristics of risk factors for constipation, such as physical activity (number of steps), diet (vegetable intakes), and mental health (K6). In the data of 5 years before the GEJE (2006–2010), the average number of steps taken and average intake of vegetables in this study area (Iwate Prefecture) were higher than the national average for both men and women.^26^ In addition, the percentage of people in good mental health in Iwate Prefecture was higher than the national average.^24^ It can, therefore, be assumed that the study area did not originally (before the GEJE) have a high rate of constipation because the risk factors for constipation were favorable compared to the national average. In contrast, this study design did not exclude those who had constipation before the GEJE. Therefore, although the effects of the earthquake are difficult to monitor, it is possible that the prevalence of constipation after the GEJE increased. Incidentally, although the study targeted four municipalities, the OR for constipation exceeded 1 only in Kamaishi city. The difference between Kamaishi and the other three municipalities is that the survey was conducted only among temporary housing residents in one area (as of September 26, 2011).^22^ This peculiarity may have contributed to the abovementioned result.

The ORs for constipation were significantly decreased after 2016 (the 6^th^ survey) in women. Furthermore, within 9 years of the follow-up, the associations between lifestyle factors and constipation were consistent. Previous studies evaluated constipation in shelters in the early phase of the disaster.^5^ However, this is the first study to assess the medium- to long-term persistence of constipation. When constipation persists for a long term, the quality of life of such individuals worsens.^3^ This is a serious concern for survivors, whose quality of life has already been compromised by living through the disaster. In addition, in recent years, concerns have emerged about the association between chronic constipation and increased mortality.^27^ Despite no associations with colorectal or any gastrointestinal malignancy, constipation may be a surrogate for general health status.^28^ Therefore, reducing the risk factors of constipation is essential for improving survivors’ quality of life and health status.

### Association between lifestyle factors and constipation among survivors

Lifestyle factors have deteriorated among survivors after disasters.^7^^–^^10^ Moreover, we found an association between worsening lifestyle factors and constipation. One reason for the higher OR of constipation in the survivors with poor mental health could be increased bowel tension due to autonomic nervous system disturbance.^16^ Moreover, poor physical activity may weaken peristalsis, increasing the risk of constipation.^16^ In women, the quantity of diet (frequency of meal) was associated with constipation in this study. Notably, reducing the frequency of meals can lead to a reduction in water and energy intake. Previous studies have reported that low water intake from food is associated with an increased prevalence of constipation.^17^^,^^29^ In fact, lower energy intake was associated with a higher prevalence of constipation in women, but this association was not reported in men.^14^ Regarding the association between a prudent dietary score and constipation, the fact that the OR was very close to 1 in our study suggests that further studies are needed to validate our result. Further, sex differences can be attributed to the effects of female hormones. Increased secretion of progesterone relaxes the plain muscles of the bowel, resulting in decreased peristalsis.^30^ Moreover, a review of epidemiological studies of chronic constipation found strong evidence that constipation is more common in women than in men.^27^ Furthermore, it is possible that women were able to provide more accurate responses about meals because women in Japan do more housework, such as meal preparation, than men.^31^ In fact, a Japanese study reported that energy intake was underestimated more commonly in men (16% in men and 6% in women).^32^ Therefore, it is possible that there was no association between constipation and meal frequency because many men ate more food than they reported.

In contrast, the present study cannot deny the possibility that the prevalence of constipation might have exacerbated lifestyle factors because of repeated cross-sectional observations. For example, loss of appetite caused by bloating, one of the constipation symptoms, may have reduced dietary intake. Therefore, lifestyle factors and constipation were possibly interrelated. Lifestyle factors worsened while physical activity, mental health, and diet interacted with each other among the survivors of disasters,^7^^,^^8^^,^^33^^–^^35^ particularly in survivors evacuated to temporary housing and public recovery housing.^8^^–^^19^^,^^35^ Appropriate measures are needed to prevent the interaction between mental health, physical activity, and diet from becoming a vicious cycle in evacuated survivors in temporary or public recovery housing.

The social connection may break this vicious cycle of this interaction between lifestyle factors and constipation. The deterioration in physical activity, mental health, and diet among survivors was associated with a lack of social capital and social networks.^8^^,^^9^^,^^33^ Particularly, survivors who moved from prefabricated temporary housing to public reconstruction housing suffered social isolation.^36^ It was expected that moving to public reconstruction housing promoted self-reliance among survivors, but in turn, social support by volunteer groups and/or local governments might be weakened. In order to break the vicious cycle of interaction between lifestyle factors and constipation, it is important to strengthen the social connections of the survivors over the medium- to long-term, even after movement from emergency shelters to temporary housing and public reconstruction housing.

### Limitations

The present study had several limitations. First, selection bias cannot be denied because the study population included those who were able to participate in health checkups for a 9-year follow-up. To address this bias as much as possible, we analyzed the participants’ data with at least baseline data, not limited to those with complete data for 9 years. Second, the cross-sectional design cannot infer causal relationships from the result. Third, the questionnaire on dietary intake did not inquire about the specific types of food or portion size. Therefore, we could not examine the association between the type or amount of dietary intake and constipation. Fourth, the questionnaire did not inquire about fluid intake and medications associated with constipation. Hence, we could not examine the association of insufficient fluid intake, laxative use, or side effects of medications with constipation. Finally, the question about constipation was not objective, since participants reported only subjective symptoms. It was also one of 25 items for multiple responses and may have led to underestimation.

### Conclusion

The present study indicated that the prevalence of constipation was the highest at baseline and remained approximately 5% in women. Lifestyle factors were associated with constipation among the survivors in a 9-year follow-up after the GEJE. Poor mental health and physical inactivity were associated with constipation in both men and women. Especially in women, a lower frequency of meals was associated with constipation. To reduce the risk of constipation and improve survivors’ quality of life and health status, this study suggests the importance of strengthening the social connection and breaking the vicious cycle of the interaction between lifestyle factors and constipation. Furthermore, our findings suggest that it is important to support the survivors in the medium- to long-term, even after moving from emergency shelter to temporary housing and public reconstruction housing.
